# Qualitative perspectives on COVID-19, interpersonal violence, and interventions to improve well-being from adolescent girls and young women in Kisumu, Kenya

**DOI:** 10.3389/frph.2023.1236588

**Published:** 2023-12-01

**Authors:** Ruby E. Reed, Mevis Omollo, Isdorah Odero, Eucabeth Awuonda, Peter Ochere, Ken Ondeng’e, Jennifer L. Kang, Jonathan Altamirano, Hellen C. Barsosio, Clea Sarnquist

**Affiliations:** ^1^ School of Medicine, Stanford University, Stanford, CA, United States; ^2^ Center for Global Health Research, Kenya Medical Research Institute, Kisumu, Kenya

**Keywords:** COVID-19, adolescence, gender-based violence, mental health, trauma, qualitative, pandemic, global health

## Abstract

**Introduction:**

Adolescent girls and young women (AGYW) face a high burden of gender-based violence (GBV) worldwide. The COVID-19 pandemic and associated policies led to global increases in GBV, decreased access to resources, and disruptions of pathways to care. We aimed to understand the effects of COVID-19 on AGYW affected by GBV in Kisumu, Kenya, as well as to identify possible interventions to mitigate those effects.

**Methods:**

Focus group discussions (FGDs) were conducted with AGYW aged 15–25 with a history of exposure to GBV. AGYW were split into age-matched groups; aged 15–19 for younger groups and 19–25 for older groups. Discussions focused on how COVID-19 affected experiences of GBV, access to care services, economic and social outcomes, and opportunities for interventions to mitigate negative impacts of COVID-19 and violence.

**Results:**

Five FGDs with 46 AGYW were completed in June-September 2021. AGYW described increases in all types of GBV, particularly sexual abuse and intimate partner violence. Early marriage and subsistence transactional sex also increased. AGYW described violence as both a cause and effect of poor economic, social and health consequences related to the pandemic. Notably, AGYW emphasized stress, lack of mental health support and increased substance use as risk factors for violence, and discussed the deleterious mental health effects of violence—particularly in the wake of disruption of mental health services. COVID-19 disrupted referrals to violence-related services, and reduced access to both medical services and psychosocial services. AGYW believed that interventions focused on improving mental health as well as economic empowerment would be the most feasible and acceptable in mitigating the negative effects of COVID-19 and related exacerbations in violence.

**Discussion:**

AGYW reported increases in almost all forms of GBV during the pandemic, with related exacerbation in mental health. Concurrently, AGYW endorsed decreased access to care services. As there is no evidence that violence and mental health challenges will quickly resolve, there is an urgent need to identify and implement interventions to mitigate these negative effects.

## 1. Introduction

On April 5th, 2020, António Guterres, Secretary General of the United Nations, called on governments to address “the horrifying global surge in domestic violence” and to put the safety of survivors of gender-based violence (GBV) first in their response to COVID-19 (1). Both direct and indirect pathways link COVID-19 and violence, including being forced to stay at home with an abuser, increased stress due to factors such as loss of income or uncertainty, increased substance abuse, and impeded access to care and support services (2, 3). Even before COVID-19, GBV could already be described as a pandemic; an estimated 1 in 3 women worldwide experience physical or sexual violence in their lifetime, primarily from an intimate partner (4). Furthermore, half of children in Asia, Africa, and Northern America experienced past-year violence (5). In Kenya, prior to the pandemic, one study found that 76% of surveyed 18–24-year-olds reported that they had experienced sexual, physical or emotional violence prior to age 18; 32% of females aged 18–24 reported that they had experienced sexual violence in childhood, and 66% of females 18–24 had experienced physical violence in childhood (6). Both experiencing and witnessing violence is directly linked to negative physical and mental health outcomes among women and children (7). In areas where HIV is endemic, GBV, HIV and mental illness have been called the “triple epidemic of global proportions,” with each epidemic fueling both the transmission and negative effects of the other (8).

In Kenya, the implementation of COVID-19 mitigation measures, such as dusk-to-dawn curfews, school closures and shelter orders, coincided with an increase in violence. At the beginning of the pandemic, Kenya's national GBV hotline saw a 301% increase in calls, and the National Crime Research Center reported a 87.7% increase in reports of GBV (9). United Nations Women conducted SMS-based surveys of 2,482 individuals across Kenya, and interviewed key informants working in fields related to GBV. They identified increases in physical violence, sexual harassment, denial of resources, emotional violence, female genital mutilation (FGM), child marriage, and sexual violence. This study also identified challenges to reporting GBV due to restrictions on movement and being stuck at home with perpetrators (10).

Some evidence has suggested that the pandemic has shifted the demographics of who perpetrates and experiences violence. At the beginning of the pandemic, Kenya saw the average age of child victims of sexual violence decrease; pre-pandemic, the average age of reporting children was 16, whereas a report during the pandemic found that the average age of reporting victims was 12 years old (11, 12). Research reviewing clinical and forensic medical documents has affirmed this finding, with data suggesting a majority of sexual GBV survivors were below 16 (13). Pandemic-related restrictions on movement and increased socioeconomic need has created greater opportunity for perpetrators to access adolescent girls and young women (AGYW), and placed AGYW in situations of greater vulnerability. Prior to the pandemic, 16% of sexual GBV in Kenya was perpetrated by non-family members; evidence suggests that this number increased to 42% after the pandemic restrictions’ onset (11–13). 76% of pandemic sexual violence cases occurred in daytime, when children would previously have been in school. Pandemic offenses mostly occurred in private locations (71% vs. 24.5% pre-pandemic), and were most commonly at the perpetrator's home (65% vs. 14.9% pre-pandemic) (11–13).

Other studies have shown a more hopeful picture of how the pandemic has affected GBV and help-seeking. A cohort of AGYW aged 15–24 in Nairobi, Kenya were surveyed pre-pandemic and then twice amidst the pandemic. Focus groups and interviews were also conducted with some participants. Results showed that intimate partner violence (IPV) rates remained stable at 17% across time points, and that help-seeking amongst the cohort increased from 11.1% for IPV and 4.6% for sexual violence in 2020, to 21.7% and 15.1% in 2021. Focus groups suggested that despite the stable rates of violence in this study, pandemic-related socioeconomic stressors and curfews were perceived as major drivers of violence (14).

Despite calls for more research and intervention into the experiences of AGYW and on GBV during the pandemic, such action has been limited. Much extant research has been focused in large urban areas; in Kenya, a majority of this research has focused on Nairobi and its surrounding communities (11–13). Qualitative studies have been limited in size, limited by format (such as having only virtual engagement), or have been coed, which may influence AGYW responses (14). Tailored interventions for AGYW in underserved regions globally are urgently needed, but the unprecedented nature of the pandemic and pandemic recovery means that the first step must be understanding AGYW needs to ensure that interventions will be effective and acceptable.

This study sought to understand the experiences of AGYW in Kenya during the COVID-19 pandemic. Specifically, we hoped to understand how COVID-19 had affected AGYW's experiences related to GBV, their access to care, and desired interventions within the community. Our goal was to collect data to inform the development of trauma-informed interventions that can mitigate the effects of increased violence. Ideally, this research could not only assist in the development of interventions locally within Western Kenya, but could contribute to knowledge about responding to GBV amidst epidemics and other humanitarian crises more generally, and provide information that may be transmittable to other communities in Kenya, Africa, and globally.

## 2. Methods

### 2.1. Setting

This project relied on a partnership between Stanford University School of Medicine, the Kenya Medical Research Institute (KEMRI), and various health center partners of KEMRI. This study took place in and around Kisumu, Kenya. This area was chosen due to high rates of HIV (15) and violence against women and girls (16, 17) including high rates of child marriage and early sexual debut relative to national and global averages.

### 2.2. Research team

Our research team was composed of Stanford Medicine faculty members, staff, and a medical student, KEMRI staff, and medical students from Maseno University hired as research assistants. All staff participated in all parts of the research, and all contributions of the Stanford team were thoroughly reviewed by KEMRI staff for acceptability and feasibility. Coding and analysis were conducted collaboratively.

### 2.3. Participant recruitment

Participants were recruited from peri-urban communities in and around the city of Kisumu, Kenya. Participants were required to speak English, KiSwahili or Dholuo, identify as female, and be 15–25 years old. Although our group was interested in including perspectives of younger girls, 15 was selected as the lower bound on age range due to concerns about capacity of younger age groups to speak thoughtfully about potentially feasible and acceptable interventions, as well as ethical concerns surrounding ability to assent to participate. This age range was also chosen due to an interest in including both married and unmarried adolescents, since those represent different risk groups for GBV. Finally, while some evidence indicates that younger groups were at an increased risk for violence during the pandemic (11, 12), this slightly older age group remains at extremely high risk. Members of the Youth Community Advisory Board at KEMRI were connected with adolescent health clinic peer leaders and nurses at Jaramogi Oginga Odinga Teaching and Referral Hospital (JOOTRH), Gita Sub-County Hospital, and a community-based organization focused on youth mental health, Tinada Youth Organization (TIYO) to educate the youth about the project and inclusion criteria. Sampling was purposive, with relevant staff at JOOTRH, Gita and Tinada identifying AGYW actively receiving services at their clinics and organizations who had directly experienced gender-based violence personally, or who had indirect experiences with gender-based violence (e.g., witnessed domestic or other gender-based violence, or sought support due to peripheral exposure to friend or family experiencing GBV). JOOTRH, Gita or Tinada staff obtained verbal consent (or verbal assent and parental consent for AGYW younger than 18). Once AGYW provided verbal consent, KEMRI staff met with participants (and parents, if under 18) to conduct the formal, written consent process.

### 2.4. Study design

We conducted the focus group discussions (FGDs) with AGYW to explore how the pandemic and related social, political, and economic changes had affected their lives. The FGDs were conducted by female study staff and research assistants from the Kisumu area who were trained on the protocol, focus group facilitation, and in the protection of human research subjects. In discussions with local team members and staff, FGDs were chosen over interviews as local staff suggested that they would likely be more acceptable to the study population of AGYW. In particular, they were expected to be more fruitful in brainstorming and discussing effects of the pandemic and ideas for interventions—a major goal of the FGDs. So as to protect privacy and avoid re-traumatization, FGDs scripts were constructed so as to encourage sharing about community problems and needs without directly querying AGYW about their own experiences of violence. Pilot testing was conducted and FGDs were found to be acceptable for AGYW.

**Figure 1:**
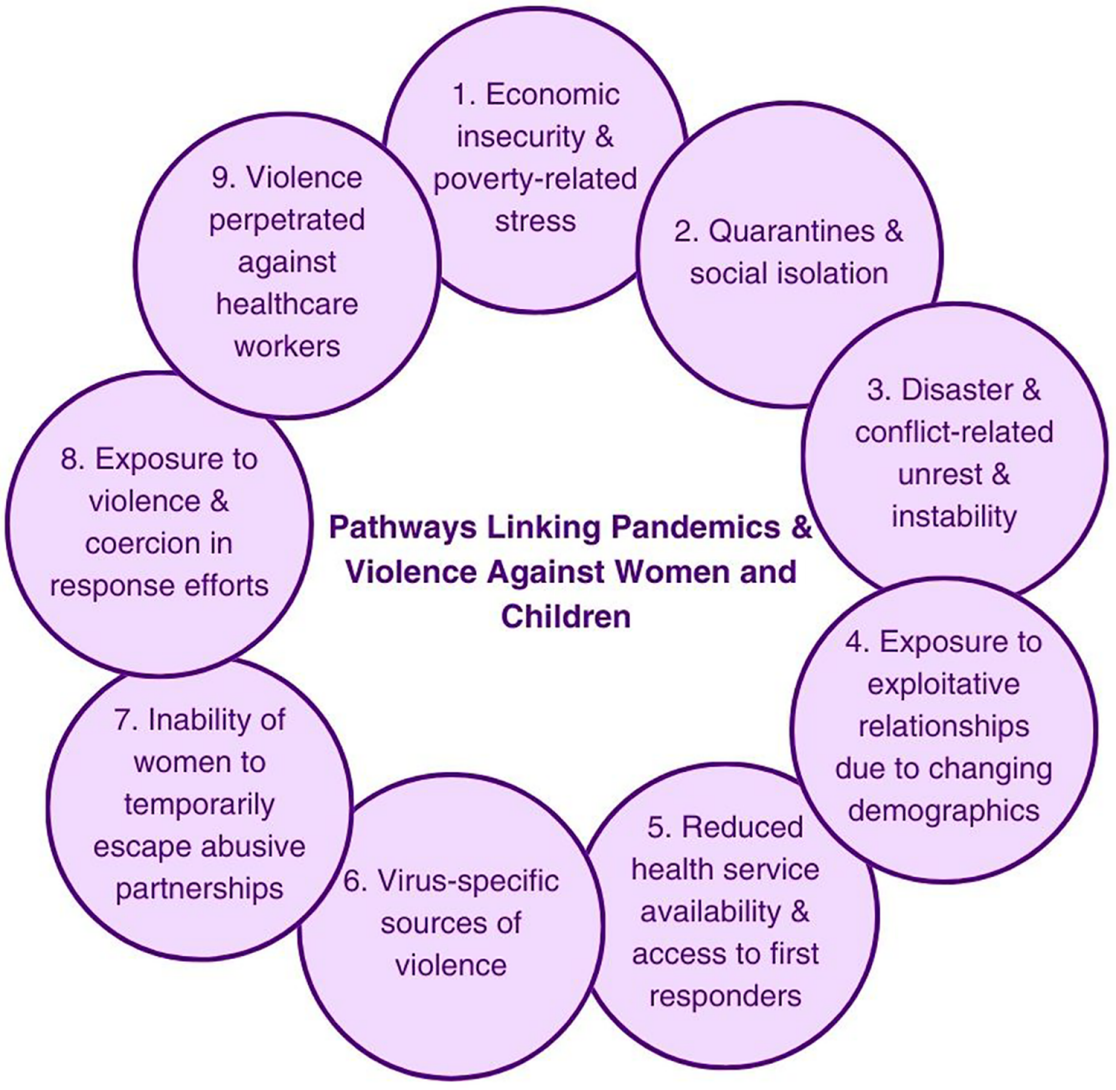
Pathways linking pandemics and violence against women and children, replicated with permission from O’Donnell et al. 2020 (2, 3). In this paper, the authors describe this figure and model as follows: “Pathways can be both direct and indirect, and are likely to interact, reinforcing existing vulnerabilities (inequalities). Pathways are not exhaustive and will depend on type of pandemic and contextual factors, including underlying gender norms and levels of VAW/C” (2).

FGDs were held in a private space at participating clinics and organizations using a semi-structured discussion guide. The focus group guide was developed based on the socioecological model of health—a commonly-used framework that describes how a vast interplay of individual, interpersonal, community, and societal/sociocultural level factors work together to cause complex public health issues like violence (18). Questions were also developed from the “Pathways Linking Pandemics and Violence Against Women and Children Model” (2, 3). As outlined in Figure 1, this model describes how during pandemics, nine “pathways” interact and both directly and indirectly exacerbate pre-existing vulnerabilities for women and children, leading to violence. Example pathways include economic turmoil, disruptions in care pathways and services, and isolation caused by disease control measures, as well as others (see Figure 1) (2). The pandemic period was defined as when the lockdowns began in Kisumu. Questions focused on the life experiences of the respondents before and during the COVID-19 period, referral services, COVID-19 and community, COVID-19 and GBV, experiences of AGYW in seeking care, and barriers and facilitators of seeking care.

AGYW were also asked about what sorts of interventions they felt would be helpful for AGYW in their community right now. They were asked to identify any successful programs they had seen in their community that had helped AGYW. They were specifically asked about programs that helped prevent violence and programs that stopped violence when it was already happening. AGYW were also asked to generate new ideas about what sorts of programs they would like to see in their community, and what these programs might look like. Then, specific evidence-based interventions that had been implemented with similar age groups in similar settings were explained to the AGYW participants, and AGYW were asked to provide feedback on their feasibility and acceptability. These interventions were chosen based on understandability to AGYW with varying levels of education generally and about GBV (i.e., concrete rather than largely conceptual), likelihood to be acceptable/feasible within the Kisumu community given local staff previous experience, and having a strong prior evidence base. The explained interventions and summary of AGYW feedback are in Table 1. After focus group 1, the “Trusted Persons” intervention was added as a potential modification to the CHV program, as this came up repeatedly in focus group 1. Demographic data about participants were collected as part of each FGD. Participants were asked to indicate their age, schooling level (“Primary,” “Secondary,” or “College/University”), marital status (“Single” or “Married”), and employment (fill-in-the-blank). Due to technical difficulties, this survey was not distributed at the JOOTRH focus groups, and girls instead provided their age and schooling level on a piece of paper; marital status was not collected. Other demographic information was collected initially, but survey questions were iterated; for example, religion was queried as a multiple-choice question in 3 focus groups; all 24 respondents selected Christian. This question was removed in further surveys.

**Table 1:** Proposed interventions and feedback from AGYW participants.

Intervention name	Description	Pros	Cons	Suggestions
Friendship Bench	Zimbabwean program where lay community members are trained to deliver mental health interventions/ counseling at a designated “bench” within the community. The intervention would be adapted to be trauma-informed to deliver care to violence survivors.	Place to share concerns/get counseling Support in making decisions Could deliver group services; provides a place for group meetings “Favors the language of the community” Peers and near-peers sometimes share better/more relevant advice	Privacy concerns (especially if bench becomes gathering place) “Advice” might be bad Adults staffing bench may misunderstand AGYW AGYW are afraid or too stressed to share things if they are identifiable	“Set rules and regulations” (ex., confidentiality) Having young adults/adolescents or a mix of adults and adolescents staffing bench Ensure individual services available and privacy protected within individual meetings Sensitize community Create method for anonymous submissions of problems, which counselor can then bring up in group discussions
Community Health Volunteers (CHVs)	A frequently-employed intervention worldwide, CHVs are trained community members who would educate communities about health issues and serve as community referrers. CHVs would be trained to educate, screen and refer for violence.	Can “share problems freely” and expect help given CHV’s knowledge and training CHV’s membership in a community and acquaintance with adolescents instills accountability	Privacy concerns, particularly given status as community member “Advice” might be bad Not all CHVs seem to be high-quality or to learn from their training, some “have an attitude and are difficult to approach” Current CHVs in communities are biased in their selection of who to provide services to due to pre-existing community knowledge If CHVs live outside their target communities, they may not visit often enough Not all CHVs viewed as “willing to help” Prior violations of trust with existing CHVs in communities	Choose CHVs who are trustworthy and respect confidentiality Good training important Appoint CHVs from different localities so as to avoid bias/privacy issues Consistent after-training follow-ups, update trainings/ reminders Train new CHVs, not existing CHVs
Hotline	A hotline that survivors could call and/or text to anonymously receive referrals or brief counseling. This may be a new hotline, or improving existing training on existing hotlines.	Increased use, willingness to share, and trust in services provided due to confidentiality Helpful for gaining knowledge and connecting with referrals No need to report to friend, family or neighbor to get help accessing care Helpful for AGYW who do not have money for travel Speed of service access	Distance from violent situation would reduce ability to provide appropriate and prompt services “Advice” might be bad, especially because of lack of familiarity Line may be busy/have a wait Fear conversation is being recorded Hard for illiterate people to learn about/use “Rude responders” Language barriers (especially for Dholuo speakers)	Ensure it is free, including for people without phone credit Ensure presence of a method to arrange face-to-face counseling for callers Polite, well-trained responders Ensure language match of responder with caller
Trusted Persons (modification to CHV protocol)	Train people trusted by AGYW – such as guidance counselors at schools, mentors at safe spaces, or other community leaders to deliver educational interventions and be community referrers for violence resources	Trust in confidentiality Trust in desire to help AGYW Trust that they will absorb training well Familiar with AGYW problems May have access to AGYW in day-to-day work		Good training important

Our plan was to conduct six age-matched focus groups, three with girls (age 15–19) and three with young women (age 20–25), or to stop when saturation was reached.

### 2.5. Data analysis

All focus group discussions were audio-recorded. Digital recordings were then transcribed, de-identified, and translated into English (as-needed) by trained study staff. Deidentified transcripts were reviewed and analyzed using NVivo qualitative data analysis software, versions 11 and 12 (19).

Data were analyzed thematically using a deductive approach, with codes based upon the Socioecological Model and Pathways Linking Pandemics and Violence Against Women and Children Model (20). Codes were initially developed based around the same theoretical models which guided questions, and were modified, deleted, or added based on emergent themes. At least two researchers coded each transcript independently, and then met to reach concordance on codes, discuss modifications to codes and emergent themes, as well as to identify larger categories, constructs, and illustrative quotes. When discrepancies or disagreements arose, a third researcher was brought in to review and discuss. Findings were also presented regularly to the full research team as an additional check on validity in the context of cultural norms and field realities. Once all transcripts were coded in this manner, the initial two researchers re-read all the transcripts to ensure consistency of coding and that no key themes were missed.

### 2.6. Ethics

This study was reviewed and approved by the Stanford University Institutional Review Board (#57394), Kenya Medical Research Institute's Scientific Ethics Review Unit (# 4103) and the Kisumu County Jaramogi Oginga Odinga Teaching and Referral Hospital Independent Ethics Review Committee (#429/21). The researchers also received permission from the Kenya National Commission for Science, Technology, and Innovation to conduct this study. Study protocols and focus group guides were developed based upon World Health Organization and U.S. Centers for Disease Control and Prevention guidelines for researching violence against women and children (21, 22).

AGYW included in the study were already connected with care services for GBV. FGDs were organized through organizations that provide care and support for GBV survivors, making care easily accessible for AGYW participants. In order to avoid revictimization or retraumatization, FGD scripts were not written to promote personal disclosure of trauma or violence, but instead to allow for sharing of opinions about needs and experiences of AGYW within the community generally.

## 3. Results

### 3.1. Participants

Five FGDs were completed in June-September 2021 with 46 AGYW, 42 of whom responded to the demographic questions (Table 2). Participants were split into five age-matched groups. One community partner, JOOTRH, operated a combined older plus younger group for AGYW that consisted of mostly older AGYW (10 total, ranging age 19–24), while the other organizations operated groups for younger AGYW (9 younger AGYW at Gita and 8 at Tinada) and for older AGYW (9 older AGYW at Gita and 9 at Tinada). Focus groups were planned to be in the primary language of preference of participants; however, due to the multilingual nature of the region, many participants spoke multiple languages during a single group session. Languages spoken within groups were as follows: Gita younger group (English and Swahili), Tinada younger group (English, Dholuo and Swahili), JOOTRH older group (English and Swahili), Gita older group (Dholuo and Swahili), Tinada older group (English, Dholuo and Swahili).

**Table 2:** Participant characteristics for girls who completed the participant survey by age group compared to Kisumu County DHS age brackets (*n* = 42, 91%, 4 AGYW did not respond to the survey).

	Adolescent girls (15–19, *n* = 25, 43%)	Young women (20–25), *n* = 17, 57%)	Kisumu county averages (2011) Women aged 15–49, weighted averages (*n* = 1,057)
Educational attainment			
No education	0 (0%)	0 (0%)	132 (12.5%)
Some or all primary school	3 (12%)	0 (0%)	630 (59.5%)
Some or all secondary school	19 (76%)	10 (59%)	296 (28.0%)^a^
Some or all university	2 (8%)	6 (35%)	
Declined (*n*)	1 (4%)	1 (6%)	N/A
Marital status			
Single	20 (80%)	6 (35%)	235 (22.2%)
Ever Married	1 (5%)	4 (24%)	822 (77.8%)
Not Queried	4 (16%)	6 (35%)	
Declined	0 (0%)	1 (6%)	N/A

Rounding may result in percentage values >100%.

^a^ Data not divided into separate secondary school and university categories when collected.

The average age for older groups was 20.9, with a range of 19–25 (with one 25-year-old included, likely consented at 24), and 17.4 for younger groups, with a range of 15–19, with the exception of one 22-year-old who joined the younger Gita group by accident. Among the younger AGYW, only 1 (5%) was married compared to 4 (24%) in the older group who responded to the survey question about marital status; however, the older group had a larger proportion of participants who were not asked this question (*n* = 4, 16% vs. *n* = 6, 35%, respectively). 3 (12%) of the girls in the younger groups had finished some or all of primary school; 19 (76%) had finished some or all of secondary school, 2 (8%) had completed some college, and 1 participant did not report schooling status. Similarly, one participant did not report schooling status in the older group, and none reported only a primary school education. 10 (59%) reported they had completed at least some of secondary school, and 6 (35%) reported had finished some or all of university education, 1 (6%) declined to answer.

Overall, our participants were more educated and less likely to be married than expected of the overall population of Kisumu. The average enrollment of girls in primary school in Kisumu District was 11.4% in 2016, with a 76.7% gross enrollment rate and 58.6% net enrollment rate of girls in secondary school (23). Further, 22.9% of girls in Kenya are married before age 18, and this number is generally higher in Kisumu (24). Given our sampling methods required youth to have agency and resources to seek healthcare or health services in an urban or peri-urban area, our sample may be more socioeconomically privileged than the general population. Most general Kisumu and Kenya statistics are based on DHS surveys conducted in 2010–2016; thus, time effects may also play a role. Our sample is also primarily urban, and excludes the large rural population of Kisumu County, which may account for some of these differences. However, the COVID-19 Gender Assessment found 15% of urban-dwelling women or girls had experienced child marriage during the pandemic; thus, our participants' rate of marriage compared to the overall population is likely low (10).

### 3.2. Qualitative findings

In discussion of qualitative findings below, findings that were consistent across groups and speakers may include short quotes that are not labeled with groups or demographics. For longer quotes, quotes are labeled with demographic information of the speaker that indicates the following: 1) location of the group that they participated in (G for a group at Gita, T for a group at Tinada, and J for the JOOTRH group), marital status of the speaker (S for single, M for married, D for declined), and age in numbers (or D for declined). For the 4 participants who did not respond to the survey, their responses are categorized within in-text attributions as “declined.” Thus, a quote may be labeled GS16 to indicate a 16-year-old, single participant from a Gita group was the speaker of the statement. Notably, all JOOTRH participants received a version of the survey without the marital status question; thus, all JOOTRH labels are simply J and age.

Quotes were attributed to specific participants by note takers during focus groups. Due to various issues during groups (ex., cross-talk, participants speaking softly, participants with similar voices), some quotes were not attributed to specific participants. In this case, quotes are labeled with the group letter and U for unknown.

#### 3.2.1. Pandemic experiences of violence

##### 3.2.1.1. Intimate partner violence (IPV)

Almost universally, AGYW believed that IPV had increased during the pandemic. AGYW largely spoke about increased physical and sexual abuse by partners; emotional abuse was not directly discussed as frequently, although some vaguely discussed such ideas as “cruelty” of partners (GDD). There were various theories as to why increases in violence had occurred, but many centered around economic factors affecting the partner, such as unemployment and stress due to finances. Whether they lived with parents or were themselves heads of households, AGYW reported considerable pandemic-related losses in income, with “no money back at home” due to lost jobs or fewer hours (TS20). AGYW discussed how they felt the economic fall-out from COVID-19 had brought about stress and a feeling of helplessness that had triggered anxiety, depression, and poor mental health. Some partnered AGYW reported financially controlling behavior in their partners that worsened during the pandemic. Numerous participants reported that they felt partners were more reticent to share funds amongst the family. For example, one participant reported that during the pandemic, her husband had begun refusing to contribute money to buy materials for their family chip stand business. Due to the pandemic, this participant noted, “there's no profits that you get, and you get to be stressed that you should just quit the business, and leave him to fend for you. That is the stress” (GM23).

AGYW also linked economic stress to poor mental health and substance abuse among their male household members, which, in turn, led to increases in violence:

There is lack of money due to COVID-19, and this has brought about depression; about how you will take care of your family, how will you pay your rent, how will your children go to school. So, when it is a man that is depressed, when he goes back to the home stressed; he will vent out on the wife by beating her and bringing about domestic violence. (TS19)

Economic stress also meant increased fighting and arguments within relationships:

[The pandemic] has caused us to despise each other in the house. Because COVID-19 has made us lose our jobs … I have lost my job and he's equally lost his job and we're just indoors. Maybe he gets a job to do for 30 min and then he comes back to the house. So, we are just seated in the house making all stories possible until it gets to a point, you're like, “Can't you even go and look for a job?” So, that feeling of despising each other creeps in. You ask for money for food, and he tells you that he doesn't have it. You're like, “How do you not have? Yet your fellow men are out there working and looking for money, yet you’re here.” So, like it has brought about lack of respect in the family. (GS25)

Increased time spent at home led to increased physical IPV; however, the primary type of IPV that AGYW described as increasing in relation to increased time spent at home was sexual IPV: “With COVID-19 … you are always in the house with someone … there is the sexual harassment between you two, like the man wants but the woman doesn't” (J21). Another participant stated: “The husband has lost his job, so we are with him every day all the time. If he has been violating me, he has more time to continue.” However, this participant noted that this increased sexual violence could encourage women to report violence or leave abusive partners: “It forces me to admit that this is now too much, so it forces some marriages to break” (GS25). However, women spoke of leaving relationships or divorce as difficult from a financial perspective:

Another thing that makes us young mothers not report is that we are helpless. Say you have two children and you have nothing at hand. Should you report, you dread a divorce, and surely if you look at yourself, you have nothing. So, you'll just persevere. (GDD)

This is notable, given participants almost universally discussed financial strain and losses during the pandemic.

AGYW identified IPV as being highly stigmatized in their communities, and it was conceptualized as a failure of the woman if it led to the failure of the marriage. Perhaps relatedly, many AGYW spoke in hypotheticals and in the second person when they spoke of IPV, despite describing specific situations. Interestingly, AGYW who had been married young near the onset of the pandemic seemed more willing to discuss having struggled in their marriages and even experienced violence. For example, one AGYW mentioned she had married at a young age before COVID-19, and reported that she had felt pressured by peers into marriage, and that domestic violence was bad within the relationship. She explained,

Say I have been undergoing violence at my home, the COVID-19 is now here, and the husband has lost his job, so we are with him every day all the time. If he has been violating me, he has more time to continue, so it forces me to admit that this is now too much. So it forces some marriages to break. And maybe I am still young, divorced from my first marriage. (GS25)

However, this AGYW stated that if violence were to recur in her life, “personally, I won't go and report.” She felt like she could not report violence to police due to “fear of what the community would say about me and our marriage,” and would instead report to members of her partner's family or the chief (GS25). As she explained:

I report that my partner has violated me or beaten me, to the police or to the chief, and then we are summoned. When I come back people will be like, “so they pretend to be in love when she is actually beaten in her house.” Or I go back to my place because he may tell me he no longer wants to see me again in his house. Then people will say, “it is a woman that builds the home, why did she escape? She should just have stayed at her home. How many women are beaten out here?” That is what makes us stay even with violence, because after all, we came to stay. (GS25)

Perhaps due to this potential rebuke in public cases, participants noted a preference for seeking care from non-formal services, such as family members, village elders, and chiefs, with most AGYW mentioning family as their initial source of support. However, some participants felt that violence had been normalized in their parents' relationships, and thus they would be unsupportive if they needed help: When speaking about reporting intimate partner violence, one participant stated that she felt that parents would not provide support: “You cannot report, you could discuss the issues with a friend. Because if you report to your parents, they will tell you that they were also beaten” (GDD).

Increased early marriage during the pandemic was seen as a driver for increased intimate partner violence. Some AGYW attributed this violence to girls being unprepared for marriage in the sense that they were not yet educated in the proper, culturally expected ways to perform as a wife: “You find that girls in the school-going age resort to marriage due to poverty. Some get into marriage without even knowing how to cook or perform other household chores, so the husband would keep beating her” (TS16). Another participant commented:

[During COVID-19] parents are taking dowry earlier. So, you are booked and you don't know that you are booked. So, you will be forced to engage in the early marriages so that the parent can get support from the person who has married you. Because they are not financially stable; so, this brought stress to a lot of girls because they felt that they were being forced with what they don't want. You've not agreed to it, but it will force you to. This was causing violence and it is still going on even with the violence it causes. (TS18)

##### 3.2.1.2. Witnessed violence

For those living with parents or other family, the pandemic meant greatly increased exposure to violence within the family, especially between parents. This violence was not entirely new, but there was a sense that “COVID-19 has made violence bitter” (TS16). AGYW reported witnessing parental violence as a major challenge for mental health and school success.

AGYW tended to attribute violence from family members as related to the economic fallout of the pandemic:

[Violence] has increased. You find that most men are always cruel when they are broke. Something small irritates them, my dad for instance. He gets very irritated when he is broke, especially to us girls. A slight mistake and he is mad at you … He is overly strict towards girls. (GDD)

At times, AGYW saw depression/stress and/or substance use as a mediator to this relationship, where economic stress spurred depression/substance use, which in turn, led to violence. AGYW also reported “fighting” due to job loss and food insecurity. Some AGYW sympathized with their fathers, noting that since the pandemic, they did not have much of a chance to look for jobs, and thus had no source of income—after which, “He starts to drink alcohol. After that, he comes to fight with mum. So mum has stress” (GS17). Others were less sympathetic:

So now he is idling … [His friends] are drunkards. So they will influence the father and he will be a drunkard also … He will start making noise. Onge! Onge! (*A Dholuo expression loosely translated to mean “there is nothing,” a phrase commonly shouted by drunk people*). He will command and want everything without caring about the source. So there will be disagreements. (J20)

AGYW did not link paternal and male substance use just with violence against mothers, however, but felt hardship-driven substance use led to many types of violence against women and children in the household. One participant shared that since the pandemic began, her neighbor's husband, a “drunkard,” had left his family without money to buy food, and that “when this man comes back to the house, it's just violence.” (GS23)

Fathers were not the sole perpetrators of witnessed domestic violence. Some AGYW lived with or near extended family, and witnessed violence perpetrated by or against non-parental figures. In particular, AGYW discussed experiences of witnessed violence by uncles and brothers:

I can talk about my brother. He is married; you find that he comes back home early. Before COVID … he used to come back to the house while tired; just coming to the house to eat and sleep. But right now, when he's back home and notices a small mistake; violence arises. So, he has a lot of time for the violence now. (GS23)

My elder brother is equally a drunkard and abuses other drugs …When he leaves the house, if he doesn't dump his stresses on me, he dumps them to our mother. Because my mum is always by my side. So, you find that he comes and starts insulting me and then my mum will support me, then he turns to my mother too; then that turns into violence. (GU)

Despite the high level of familial violence reported, AGYW did not identify this as an area for intervention, largely due to the interdependencies of families:

You will sit down and think this is my dad, and he is the one who always provides. And I report him, and he is arrested, who will be providing for the family? So you just decide to keep quiet about it (TS18).

##### 3.2.1.3. Sexual assault & harassment by non-partners

AGYW frequently used the term “sexual harassment” to refer to rape—for example: “I can say that one of the causes of stress [for girls is] sexual harassment—like if you are a girl and you are raped by a man, you will have stress” (J20); “In terms of sexual harassment, maybe you have been raped by a man and you are at a point where you won't even remember to take PrEP [Pre-Exposure-Prophylaxis] or pills” (J20). However, overall, it is unclear whether any isolated mentions of sexual harassment refer to its standard definition, or to sexual assault or rape, and it is not possible to draw substantial inferences about sexual harassment from the data. However, this phrasing in and of itself is an interesting finding—and perhaps indicates a lack of education about sexual harassment, that “sexual harassment” is understood or experienced differently by AGYW in Kisumu, or that this terminology is not the most accurate to use within this population for what is typically defined as sexual harassment.

AGYW universally recognized that sexual abuse, especially of children, was, indeed, abuse. Participants stated it was a common problem in their communities even prior to the pandemic. AGYW linked economic need and parental substance abuse to pre-pandemic sexual abuse. AGYW placed in situations where they were vulnerable to exploitation, such as poverty, were particularly at-risk for sexual abuse:

Let's say this is your uncle, and you need food or pads from him. Let's say you ask for pads and stuff. He gives them to you, and the second time he says you must do something, so you end up sleeping with him … I have a friend who had to sleep with the uncle—the person who was supporting her—and now she is pregnant. And she is in form 2. There was no agreement, because they decided to settle it as a family matter. Now the lady was really affected, because she is form 2, no one to support her, and she can get blamed and told she is the one who went there. So there is depression there. (J24)

Interestingly, this participant referred to this event as “sexual harassment,” rather than rape or abuse. It is notable that this situation is not dissimilar to other situations described within the focus groups as “prostitution” or “relationships,” suggesting perhaps that these terms may at times be used to refer to similarly exploitative circumstances. However, these other relationships were not necessarily regarded as abusive, and outlook towards women and girls within such situations was much less sympathetic.

Many AGYW noted increases in transactional sex generally during the pandemic, which increased vulnerability to sexual assault and rape:

Young girls aren't supported or educated by their parents about sex education. So if they want something and their parents don't give them they go and get it from somewhere, let's say a man, and they might have to pay with sex, and if they don't, they get raped (J22).

AGYW felt that the economic circumstances of the pandemic were forcing AGYW into subsistence transactional sexual relationships and sexual decisions:

Most of the young women or young girls have gone into prostitution since there are no jobs, and there is no money. So, it will force her to go into prostitution so that she can meet her needs. Probably she has a young child like this one here [*references lap infant*], who needs diapers, and there is no money to buy it for her. So, it will force her to look for someone. (GM23)

Another situation that participants felt increased vulnerability of AGYW to sexual abuse was growing up in a relative's home. Like the situation described above, other girls described situations of sexual abuse when girls were “brought up by [their] aunt” or other relatives. One participant described experiencing sexual abuse by boys/men when “where you stay is not your home,” and its deleterious effects:

Even though you might be lucky not to have contracted any disease, the experience remains engraved in your mind. Even when you see any guy, regardless of how old you'll be; you don't want to associate with any man. When you see a man, it brings back bad memories. Even when a guy talks to you nicely and tries to be friends with you, or even when in school you can't sit next to boys because the experience is engraved in your mind. (TM24)

AGYW felt that COVID-19 increased their vulnerability to sexual abuse in part due to such greater opportunity for victimization. More AGYW were at home for longer periods of time, interacting more with other members of their households. Further, more AGYW moved in with extended family or non-family members; AGYW spoke about transitions where families dispersed to live in rural areas where rent was lower or nonexistent. Other AGYW shared that their parents had rescinded their support of them during the pandemic, and they were forced to find housing elsewhere—increasing susceptibility to violence.

I can say that COVID has brought problems to girls and young women, when sometimes you bring in a female relative to stay with you, your husband may abuse them sexually. The same may also happen with male relatives from the husband's side abusing the wife sexually. So, it may affect you mentally, or generally health-wise. (TM23)

AGYW in multiple groups described what they perceived as a clear causative pathway between substance abuse, mental health challenges, and sexual abuse: men lost jobs and money, this caused stress/mental illness, which led to men drinking, which, in turn, led to perpetration of intra-household sexual abuse. In particular, this causative pathway and the excuse of substance use was almost always mentioned when explaining father-perpetrated sexual abuse. In discussing stressors within her community, one participant noted:

You find that both parents were working but when one, this is the man mostly, if he gets fired then goes back home, he is the one who is always affected the most … So the dad is at home most of the time and so he gets into depression and starts to drink and once he drinks he goes fully into it. If he had an argument with mum in the morning it adds him more stress and if he has a daughter he could rape or sexually abuse the girl. (JU)

##### 3.2.1.4. Extreme economic need, child neglect & financial abuse

Across the board, participants discussed the pandemic as a time of extreme financial hardship. For the most part, this was discussed as a difficulty that AGYW faced with their families. However, pandemic-related economic issues also put AGYW at risk for neglect and financial abuse. Some participants also reported that their partners reduced resource-sharing in their households during the pandemic; financial and economic IPV was discussed in section 3.2.1.1.

Participants discussed denial of school fees as a major cause of stress and low self-esteem for school-going girls: “If your father is always like, he doesn't have school fees, he can't take you to school, so you will just feel bad about it.” (TS16). Economic fall-out of the pandemic meant increased anxiety about whether AGYW would be able to return to school due to a lack of funds for school fees: “COVID-19 has had us questioning how we will go to school. [….] there is no money—your parents are constantly complaining. You do not know how you will continue with your education” (TS19). Exacerbating the situation, many AGYW were forced to repeat a year of school due to the pandemic, which meant families incurred an additional year of fees. Lack of economic support caused strain in AGYW-parent relationships—sometimes inducing distrust, as adolescents did not necessarily believe that parents truly lacked the means to support them.

Conversations about denials of school fees were not simply about economics, and often hit on deeply emotional topics—disrupting their time in school. One AGYW shared about a difficult conversation related to paying for secondary school fees:

You might find that sometimes you are going to sit for your KCPE (Kenya primary school leaving examination) and before that, your parents have been arguing a lot, and then, there is a time your father will tell you that you are not even his child—you should go back to your father … So you can't concentrate during the exams because the words your father said are still in your head. So after failing, you might decide to commit suicide because he already said you are not his child and he can't take you to high school. (TS16)

Perceived financial abandonment by fathers also led to disruption at home, including arguing between parents and disruption in their own relationships with their parents. As one AGYW stated, “You will find a father was working and paying bills at home. So during COVID he gets little money and you also want to go to school. There is no school fees and that brings chaos in the house” (GS16). Another explained, “A father has refused to pay school fees—that can make him argue with the mother, because the mum feels like he doesn't love their child” (TS16). Even girls who reported their school fees were still being paid, but perhaps late or more intermittently, still reported substantial stress caused by this delay and the discussions around it. AGYW also pointed to lack of economic support for school as a driver of subsistence or near-subsistence transactional sexual relationships. When asked how the pandemic had affected AGYW, one participant specifically stated:

Lack of school fees will lead to school dropout and later high birth rate. When girls drop out of school, they will have the opportunity to go and visit their boyfriends. So she goes there and says to herself, there is no worries even if I get pregnant. There is no money at home so I can just stay with the boyfriend and life continues …So they will go there because the parents don't have money to take them to high school. (GS17)

[The pandemic] really affected the parents, because most of them lost their jobs and were at home … with the kids, yet they have needs … So when you go to ask your parents for money, they will tell you they don't have money. So when you go out, and you find a motorbike guy, and he offers to provide for you in return of sex …[He] buys you pads, and buys you chips worth 20 shillings ($0.15 USD) when you are hungry, and he does the same thing the next day …He will just be doing things for you, and next time, he will tell you to go to his house, and you will have sex … [when] parents don't provide for them such things, it will make them to go look for them somewhere else, and they end up being pregnant. (J24)

However, participants did not necessarily implicate willful parental abandonment or neglect as the major driver of subsistence sex. Rather, AGYW described how the pandemic worsened economic situations and outcomes for families, and their own perceptions of their parents' economic need drove engagement in transactional sex. As one AGYW stated, “You know behind the curtains you see your parents does not have money, so you go and associate yourself with bad company—like having some sponsors to support you” (TS18). AGYW noticed new, extreme situations at home where they felt their economic needs were unlikely to be met. As one participant noted:

What I'm seeing, COVID-19 is really going to affect me, because my dad right now earns so little money—of which, I have started to doubt, and asking myself: When I finish school, am I going to stay at home? Or where will I start? And where will I finish? Because I'm seeing my dad's money is reducing and he is also earning little money. (GS18)

One participant described how COVID-19 had caused numerous stressors for families, including infection with the virus itself causing fear, loss of life, and financial losses. She stated that all of this had “led to depression and that brought violence at home.” She explained the effect of reduced parental support due to stress, financial losses, or human losses due to disease:

So [COVID-19] brought a lot of challenges to young girls since they had no one to provide for them. So that led to young girls going out of their way looking for ways to survive. So let's say 99% [*pause*] 70% of the young girls got pregnant because sometimes you go to look for work and when you get paid, you feel like that is not enough, [so] then you get into a relationship and then get pregnant, depression and some even commit suicide. (JU)

Another participant explained that from her perspective, increases in transactional sexual relationships were not necessarily for subsistence needs:

COVID-19 has made a lot of teenagers to start dating. You find that a lot of people have lost their jobs and hence, a lot of parents have gone back to staying at home. And then since you are a girl, you want to dress up like your peers do. So, since your parents can't provide since they've lost their jobs; and you can't look for a job since you are underage. So, you resolve on having a boyfriend that when you ask for money, he will be able to send it to you. You get into that relationship with a teenager mindset, and you end up getting pregnant and some may leave you HIV infected. You find that even students in class 4 or 5 have started dating because of COVID-19. (TS19)

AGYW pointed to increases in these relationships as causes of rises in early pregnancy, marriage, and HIV/STIs in their community. These relationships were discussed as direct causes of low self-esteem and depression, and also as indirect causes through their causing early pregnancy, school drop-out, and HIV/STIs. As one stated,

You find your parent doesn't have a job wherever she is, so she can't provide for you what you want, or the person you were depending on can't provide for you. This will make the girl to look for a man to give her something in return for sex. And when she goes to the hospital she goes to the hospital she is told she has HIV and STIs. And that can lead to her depression and her self-esteem goes down. (J19)

In Kenya, the pandemic was associated with rise not just in school drop-out, but in early pregnancy (25). Participants noted this in their community in Kisumu as well. One participant commented on an “increased number of boy-girl relationships, which has further led to early pregnancies and sickness” (TS19). Some participants discussed how AGYW became more rebellious during the pandemic period—in part due to the highly restrictive lockdowns—which led to engagement in higher-risk relationships and sex. Others noted that with schools closed and restrictions on movement, there was little to do, which sometimes led to engagement in risky behavior out of boredom. Some participants noted that they had more time to visit boyfriends, which led to pregnancy. One participant explained of the time at the beginning of the pandemic, “I was not obeying my parents. I used to obey my boyfriend. The boyfriend made me pregnant and I started crying and I told him I’m staying with him” (GS17).

At times, early pregnancy became a driver for parental abandonment and neglect. Participants often discussed pregnant or mother AGYW as girls who “don't have parents” (GS17) or girls whose “parents maybe already gave up on her because she was misbehaving at home” (GS18). Participants explained that once AGYW became pregnant, parents often withdrew support and saw AGYW as the responsibility of their partners:

Because some of our parents are not good, the moment you tell them that you are pregnant, that's the thing that they will use to abuse you. They will tell you like this: “there is no reason for you to go to school and yet you just know how to open your legs. The best thing you can do, you just go and open your legs for those men.” They are talking badly to us, so those words they hurt us, even you don't have the courage to go to school. It's better you drop out of school and just get married. (GDD)

Challenges with parents in the face of pregnancy could force AGYW to remain in difficult or abusive partnerships. One participant suggested that pregnant AGYW may be forced into negative relationships:

In your family, your parents are strict, so you're left asking yourself on what you'll do …Sometimes you have to run away from home and gone to get married to the person who got you pregnant. It will force you to stay there even when you are suffering (TM24).

##### 3.2.1.5. Physical & emotional child abuse

Physical and emotional child abuse were the least discussed types of violence within the focus groups—although perhaps not due to infrequency within the community. Throughout the discussions, the topic of parents “beating” children was discussed in a casual manner; most AGYW did not seem to perceive beatings or harsh corporal punishment from parents as physical child abuse. For example, when asked what common problems they see in their family, one girl responded, “When you go to fetch water from the river, and you take a lot of time, when you go back home, your mother will beat you up” (GS18).

Despite the normalization of “beating,” AGYW reflected repeatedly on its negative effects. AGYW expressed a desire to escape physical abuse: “My mother … when she beats me or tells me something, my mind always tell me to run away or leave school.” Many AGYW shared reactions to emotional and verbal abuse, stating that “when you are at home and your parent likes to quarrel you every day” it can decrease self-esteem and increase stress and depression (GS18). Some AGYW indicated a strong desire to escape the situation by any means. In relation to experiences of physical abuse, AGYW expressed desires to “give up” on school. Some AGYW also commented on the heavy weight of emotional violence, stating that it can cause reductions in self-esteem and difficulties in relationships with others, as “you will just feel like everyone who is around you will abuse you” (J24). Another participant stated that emotional violence could cause them to become “depressed—like it will reach a point where you see death is the only way out” (TS18).

Some AGYW pointed to normalization of physical and emotional violence as a challenge for them reporting abuse of other kinds to their parents. As noted above in 3.2.1.1, parents were not seen consistently by participants as a safe group to report to in cases of violence, typically due to internalized norms based on their own experiences of violence. Other AGYW endorsed the idea that parents were not sources of unconditional support in other situations:

Your mother can be so strict that at times you have gone to share your problems with her but she's just there scolding you. That makes you hold onto the problems that are affecting you rather than open about them to her (TS19).

AGYW did not comment much on changes related to the pandemic and physical/emotional violence. At times, it was difficult to parse if participants were speaking about experiencing or witnessing violence, as they spoke generally about increases in “violence at home” during the pandemic without specifying who was experiencing the violence (JU). However, parents were often implied to be the perpetrators, as multiple participants explained such violence increased due to parents' stress over lost jobs and work hours.

AGYW noted deterioration in their relationships with parents during the pandemic. There was a reported “lack of freedom” granted by parents (TS18) and “lack of understanding among family members” (J20). AGYW described this disruption in their relationships as causing increased arguing and fighting at home. As one AGYW stated,

You find that most of the young adults like us, they don't get along well with their parents because they want to do things on their own, but parents also want them to do what they expect them to do…they don't understand us. (GDD)

In response to this, some AGYW reported that they felt that they or their friends had become more defiant of their parents during the pandemic: “When their parents try to talk to them, they take things oppositely from what they are told. And they don't even want to listen to what their parents are teaching them” (G19S). Some AGYW related this defiance to significant consequences, with more than one girl reporting leaving the home and becoming pregnant.

Some AGYW, however, associated the pandemic with improved relationships with their parents. As emphasized by one participant:

COVID-19 has made us to associate more with parents. Before the 7PM curfew, you could find that your father would come back to the house at midnight and leaves at 4AM. But now with the curfew, you find time to converse with him and share in stories; so that he knows the challenges that you face. (T19S)

##### 3.2.1.6. Early & forced marriage

Consistent with prior survey studies of key informants in the region (26), AGYW perceived increased rates of early marriage in their communities during the pandemic. Girls reported feeling stress and pressure from their families, peers, and general community to marry, especially in the wake of economic turmoil and school closures. One participant noted, “Maybe my age mate has already gotten married…So you’ll find that I’m being told, ‘You are not going to school, there's nothing that you’re doing; you could probably look for a man to marry you’” (GS25).

Financial hardship was reported as a major driver for increased rates of early marriage. AGYW stated that despite desires to return to school, they could not. One participant, a teacher, stated that she had seen many of her students drop out of school and get pregnant or married, despite desires to continue schooling:

Even if you want to go to school, the parents will tell you that they don't have money to take you to school and that you just stay at home until they find money …[so] instead you look for someone to marry you so that you can go and stay with them. (TM23)

Another participant pointed to fear of losing housing due to reduced parental support and economic strain:

When COVID-19 started, it made a lot of young girls to get into early marriages. In my case, I had one child, but when the COVID-19 pandemic started, I got pregnant with another child. I live with my parents, and when I got pregnant with my second child, they had a change of heart. They were asking where I was going to live with these two children. Now that is what is making us get into early marriages. (GM22)

Other AGYW spoke about early marriage as a more direct attempt by their families to remediate their financial problems. Some commented that AGYW whose parents had lost jobs, who were survivors of violence, and/or did not have present parental figures were more vulnerable to early marriage during the pandemic. When responding to another participant speaking about causes of school dropout during the pandemic, one stated: “A girl's parent doesn't have a job, and the father wants to sell her to another person so that they can get the money. So this will force her to drop out of school” (TS16).

AGYW noted that they also felt that early pregnancy increased during the pandemic, and that this was a driver of early and forced marriage. Some AGYW noted that survivors of sexual violence were vulnerable to forced marriage after sexual assault, particularly assaults that were made public or resulted in pregnancy. AGYW seemed to indicate that the increased economic need created by the pandemic encouraged a situation where early pregnancy was more likely to result in an early marriage. AGYW stated that without their own money and/or with their parents losing jobs, pregnant AGYW needed to marry to guarantee access to shelter and food. As one participant explained:

Now that COVID-19 is here, let's say someone was raped and they did not seek medical attention, and the parents have lost jobs or she is an orphan, so there is no money. It forces them to get into early marriages that were unplanned for, so that they have a roof over their heads, food, and a maybe a place to call home. (GS25)

#### 3.2.2. Desired interventions

AGYW were asked for their opinions on ideas and programs that could improve the lives of AGYW in their community. It was explained to participants that they were the experts on the lives of AGYW in their community, and thus their expert opinions were needed. AGYW were then asked about extant interventions, to come up with ideas for interventions, and to provide feedback on specific intervention ideas.

##### 3.2.2.1. Desired interventions: mental health

When discussing the challenges that the pandemic caused for AGYW, participants repeatedly discussed reduced community-based mental health and support services. AGYW commented that resources existed in their communities, but that they targeted people with other needs, ages, or genders, and that “[services] are there, but they don't help adolescent girls and young women … For us it feels like we have been abandoned” (GS25).

Prior to the pandemic, many of the participants had access to “safe spaces”—a common community intervention where young women and girls can seek information, services and social support (27). Such youth-friendly community/health centers closed during the pandemic, cutting off access to many services, including “mentors” who could provide counseling. School closures also meant that AGYW were without school-based support services or supportive relationships, such as from teachers or guidance counselors. As such, AGYW felt they had “no guidance,” and without proper guidance and counseling services, “you will find us missing our ways because there is no one to advise us” (GS17). As another participant stated, “Girls like us need to be reminded of how we should live so that we go on with our lives” (GS17). Participants specifically noted that guidance and counseling services for youth living with HIV were also closed. Lack of services exacerbated health problems these programs sought to address, but also contributed to the newfound loneliness and stress AGYW were facing:

We have mentors in our safe space, those are the people who we were meeting with and telling them what is happening in our lives. But now the challenge is, we cannot go and meet them. … So that one challenges me because I can't confide in anyone, even if I'm sick or affected I can't talk to anyone. Not just physically sick, you know, like sometimes you can go have sex with someone and you don't know his status. So like I come here and test positive for a STI, I can't go and tell my mom …That will add stress. So if go there and tell them my situation they would know how to deal with it. But now there is no one I can talk to. (J24)

When asked what services would be most helpful in reducing the effects of the pandemic on AGYW, many participants were most enthusiastic about re-starting or enhancing support and mental health-focused programs, such as support groups and counseling:

But when we sit down as ladies and everyone shares their problems, even if someone was depressed, the depression might decrease. But when you are at home and, for example, your mother wronged you, and you don't have anyone to share with, sometimes you go and lock yourself in the bedroom. (JU)

AGYW described counselors they had encountered as “motivating” and “encouraging”; one participant stated that counseling/group therapy is “not only a way of helping people, but it brings people together as friends, so it also builds a strong relationship where you can confide in anything personal” (TS18). Another participant stated,

I think one-on-one talks with these women [counselors], you get to see the challenges they are going through, it helps in the reduction of stress and depression. When you have that person to tell you it's going to be alright and it will pass, that will help. (J24)

Notably, AGYW referred to these counselors and mentors as women. Mentorship and counseling were spoken about interchangeably by AGYW, with participants expressing a need for both survivors to receive counseling and to be involved in providing it. For example:

My idea is that girls who have successfully overcome violence situations be identified, and they would be offering mentorships to the younger ones who have not undergone violence. This is because they would be talking out of experience. (TS19)

AGYW seemed to prefer that these counselors be from their own communities, and that they meet them in their communities. For example, one girl stated that her desired intervention was that, “You might find the counselor go to an area and take a few girls, meet with them and counsel them” (GS17).

Participants particularly pointed to support groups as beneficial for AGYW survivors of violence. As one participant described, group counseling sessions had existed prior to COVID, but these services had largely been eliminated since the pandemic began, and as such, “you may end up thinking you are the only one going through that challenge” (TS19). As another participant commented:

So when the [counseling program with support groups] was there, so many girls used to come. So when you share a problem there and you hear someone has the same problem as you, this will encourage you and feel like you are not alone. But now you are all alone. There are no such gatherings. So you will start thinking of how people will perceive you if you go and say your problem. (J23)

AGYW felt that it was especially important to have mental health counseling for survivors of violence; some AGYW also mentioned how this would be helpful preventively. For example, they stated that good advice and guidance from counselors would steer AGYW away from negative relationships and decisions, or could assist in secondary prevention early in violent relationships. However, participants mostly spoke of mental health support as helpful during or after violence. As one AGYW noted:

If you are raped or you have been emotionally abused, your self-esteem will reduce. Then the trauma [*pause*]*.* If you've not had good counseling sessions, the trauma will still be there. It can't disappear like that. You will just feel like everyone who is around you will abuse you. (J24)

However, opinions of mental health counselors were not universally positive; AGYW were concerned that counselors would not be sufficiently trained, would not come to the communities sufficiently often, would not speak the local language (particularly Dholuo), or would not maintain confidentiality. In conceptualizing mental health services, some participants seemed focused on “advice,” perhaps equating the two. Some participants expressed concern about specific services (such as FB or CHVs) that they may not give high-quality or correct advice to AGYW. Referring to the FB intervention, one AGYW stated,

You might share your problems there, but no one will give you an advice that will help you. They might tell you to continue with what you were doing or you leave it. So you will be given different opinions of which maybe might not help you. (GM18)

Other AGYW were concerned that when seeking mental health services, counselors would not treat survivors with compassion:

“I think when you go to a counsellor for counselling, some of them are too harsh. Then they’ll start asking how you were raped at 20, 25 years. They may claim that you are so naïve, and you are the one who went there by yourself” (TS20).

##### 3.2.2.2. Desired interventions: economic

When asked what interventions were best for primary prevention of violence, AGYW generally emphasized economic empowerment. AGYW across groups agreed that the pandemic had resulted in severe economic fallout for their families. One participant stated,

COVID-19 has affected our lives in that we currently do not have money …Everywhere that you go, you hear people complaining that there is no money. It has also affected the cost of life; things are expensive and so we feel that life is hard. (TM24)

School closures also contributed to poor health and economic outcomes for AGYW during the pandemic lockdowns due to the decreased accessibility of community services and economic goods. Community services that had helped AGYW with educational and tangible needs (such as hygiene supplies, clothing, school fees, and cash assistance) had disappeared during COVID-19. AGYW across age groups repeatedly mentioned that they had reduced access to sanitary pads (typically provided through free programs, such as at schools). This was a substantial stressor for very low income AGYW. When asked what services would be helpful to reduce the impact of COVID-19 on AGYW, one participant responded, “For me, financial support. As a young woman, maybe I am not in school so I don't have someone who will provide for me the sanitary towels” (JU).

During the pandemic, AGYW saw school-age girls in their communities forced into provider roles for their families. As one adolescent girl put it: “You will find someone my age, the parent isn't that well off … So she will force herself to do other jobs, so that she can help the mum to feed the family” (GS17). AGYW felt that those who had left school for work would be unlikely to return to school once pandemic restrictions subsided. Another participant said: “Let's say that someone is in form 4 and during COVID … she gets a job … she will drop out of school and continue with business” (TS18). AGYW indicated that economic incentives would be needed to encourage school return for girls forced into the workforce.

AGYW who had been working prior to the pandemic lost jobs and wages due to the pandemic, and struggled to find new jobs. One participant explained: “During this COVID, the earnings are low, and the investment is also low …No one will give you a job. Maybe you were fired from work, this will force you to stay home because there is no work” (GM18). Many explained that AGYW often held positions in communities that were particularly affected by pandemic restrictions, such as street vending, working in bars/restaurants, cleaning, teaching, or hospitality work. One participant explained,

“[Curfew] has greatly affected the industry that I am working in [restaurant/bar], to the extent that I had to quit my job because the owner of the business wanted to reduce the pay to half the initial salary” (GS25). As one participant reported “everybody is at home now, no money, lack of food” (GS18).

AGYW felt that economic empowerment would be a useful tool during the pandemic in relation to violence. Kenya instituted cash stipends during the pandemic for very low-income populations. However, access to this program was reported to be in uneven, with a delayed roll-out to Kisumu, and had strict inclusion criteria (e.g., prioritized participants who were living in households where heads of households were widowed, minors, had disabilities/chronic illnesses, or other criteria) (28). Some participants reported receiving some money from this program. Speaking about the program, one participant noted, “Not everybody [was receiving it] but I think women could say there was some support that even if you were violated, you had something small to start with” (J23).

Employment status was also noted to affect violence. Some participants noted that women who had been able to keep jobs—particularly when their husbands were not employed—had gained power in their relationships. One participant noted, “[Violence] has decreased for those who have lost jobs and the female partners are still at work. They [men] are then forced to bow low, because the wife is now the breadwinner, and calls shots, financially speaking” (GS25).

Pointing to their increased economic need, participants, particularly older participants, felt economic support and empowerment were crucial to improving the situations of AGYW, especially those experiencing violence. Participants felt small, one-time payments would provide large benefits—explaining that with a small parcel of land they could do farming to sustain themselves, or with just KSH 5,000 ($37 USD) they could “open a small shop for clothes and shoes or a vegetable stall, and then I’m okay because I have an income” (JU). AGYW felt this would not just improve their lives generally, but reduce their risk of exposure to and victimization from violence. As one AGYW said, “We would need financial support so as to reduce the unemployment that is there, the domestic violence that is currently ongoing. People be given money to start off businesses, the youths be employed” (TS19). Another participant noted:

At least when I have my own job, I will not be so dependent on a man, because at the end of the day, what causes arguments in the house is the issue of money. So at least when I have my own money and I have my own job, I can depend on myself. I will not be the type of wife that is constantly asking the husband for money, even for things as small as matchsticks. (GS25)

Participants also noted that non-monetary resources would benefit them, such as the provision of hygiene supplies such as sanitary pads, clothing, and books. AGYW also felt assistance paying and/or a reduction in school fees for school-going AGYW or those who had been forced to drop out of school during the pandemic was an important need in their communities. As one AGYW stated, she felt that helping AGYW recover from the pandemic would best be accomplished by “lower[ing] the amount of school fees. [The government] can also sponsor children. Like, for example, 20 students from a village. And that will make everyone who wants free education to join” (GS18).

##### 3.2.2.3. Desired interventions: education

AGYW felt that more should be done to educate the community, and especially young people, about violence—“how to deal with violence, what they are supposed to do when they come across violence” (GS17). AGYW also felt that more specific education for AGYW on their rights, sex education, and “teaching these girls to understand their bodies” would be helpful to prevent violence (J22). AGYW had different ideas about to whom and when this training should be given—proposing alternatively that school-going girls aged 9–18, young mothers, or all girls across the country should undergo these trainings. AGYW were also interested in guidance counselors or mentors who could provide them with resource referrals for health and economic community services. Participants proposed that this education should come both within formal settings, such as schools and community programs, but also through creative methods. One participant emphasized social media and radio, because “most people can afford to have a radio” (GDD). Another participant expanded on how such an intervention could succeed within the community:

I think for the youths … for education, the things like TV, every youth I can think of right now has a smartphone or a kabambe (*a very basic mobile phone*), so many are in a position to access these devices. And TVs, there are many TVs, so we can get the information there or from radios. Then from the phone we have social media like Facebook, Twitter. There are some who do not concern themselves with such though. So it can be passed through the radio or even SMS. There are these SMS that pops up in phones and you don't know where they are from. (J23)

#### 3.2.3. Feedback on specific interventions

When asked which idea would be the best to stop violence that is already happening, AGYW generally felt the Friendship Bench (FB) would best mitigate violence in their communities, with the Community Health Volunteer (CHV) trainings also frequently mentioned. Some AGYW felt the FB and CHV trainings would better prevent violence if combined. When explaining why, participants agreed that having a community-based resource and advocate who was physically present and from the community was best for stopping violence. As one participant compared:

Yes, the Friendship Bench [would be best] because CHV may have been possibly selected from another village … And so in this situation, if the CHV is not around, it will be hard to reach out to them. But for me, the immediate person who will help, I think is this Friendship Bench. (TS18)

Other AGYW stated the hotline would not work because it was too distant from violence that was actively happening. But some participants liked the CHV training idea, stating that “we need people physically on the ground to talk to and obtain advice from” to stop ongoing violence (TS19).

AGYW were also asked which intervention would be best for preventing violence. The FB and CHV trainings got near-equal support in this realm, with similar explanations: AGYW felt that education, support, and advice, as well as intervention on early problem behaviors were the best preventive strategies. Some felt that the FB was beneficial because it would provide a community gathering place and a sense of support. One participant stated,

According to me, Friendship Bench [is best]. Because, while you are gathered, some pieces of advice would flow. Another thing, if you see a bad thing is going to befall you, you could share it among your friends and get some healthy advice. (GM23)

One participant felt the FB would work well because the counselor would be relatable and may have had the same problem in their past, and “somehow your friend is experienced, and he or she can guide you” (TS20). Similarly, another participant stated, “For me I think the Friendship Bench will work because there are those who you feel you can confide in as friends. Talk to them, ‘I have this problem’” (TS18). Others focused more on the counseling itself when discussing the FB: “I think it's the Friendship Bench [that is best for violence prevention], because when we come together and have someone who can guide us, it will work” (JU). Similarly, another participant was confident that CHVs “will stop [violence] before it happens because the people that are helping are trained and would advise” (TS20). Yet another participant reported they felt CHVs would help prevent violence by recognizing violence and reporting it. AGYW felt that the training that CHVs undergo was important, and relatedly, were concerned that without this training, interventions may be less effective; for example, one participant said that CHVs were “trained and know better, and the Friendship Bench can just be for giving advice” (GS18).

Many AGYW stated that they felt that “the CHVs and Friendship Bench go together” (JU), and that they thought a program that combined elements of the FB and the CHV training would be best for stopping and preventing violence. AGYW wanted the FB to “have a leader who can look into our problems, then it can work” (J23). Many such suggestions described the planned function of the FB, which is staffed by a trained counselor or CHV. These responses indicated that our explanation was unclear, or the name “Friendship Bench” may be confusing. This unclarity may have been part of AGYW hesitations around the FB, including concerns about confidentiality, such as fears that community members may just come to the bench to hear other peoples' gossip. As one participant stated, “The Friendship Bench contains people who are just there to help, but there are some people there that you are not in good terms with so they will go and tell others” (GS17). Others had concerns that “advice” may be poor; this was a concern across all interventions. Some of the AGYW's suggestions to improve the FB were already facets of the FB programming (e.g., training of counselors), and may indicate a need for community sensitization and/or adjustment of the branding of the intervention to better fit the Kisumu community.

## 4. Discussion

### 4.1. Key findings

In this study, AGYW reported that during the COVID-19 pandemic, there were concerning increases in numerous types of GBV as well as decreased access to services—consistent with findings around the world (13, 29–31). These findings are also consistent with existing Kenya-specific findings, which suggests that sexual and intimate partner violence increased in Kenya during the pandemic (32, 33). AGYW particularly emphasized increases in witnessed physical IPV, as well as experiences of sexual IPV and child sexual abuse. As there is little evidence that issues like violence and mental health challenges and their impacts will quickly resolve, there is an urgent need to identify and implement interventions to mitigate the long-term negative effects of these issues as the world moves on from the emergency phase of the pandemic.

The AGYW in our study had also appreciated in their communities many of the negative effects of the pandemic and of violence that have been noted in other studies. Pandemic increases in violence have been associated with negative impacts on AGYW, including increased early marriage and pregnancy, and decreased financial independence (34). Similar to a qualitative study conducted of adolescent girls in Nairobi (14), our study found that pandemic-related restrictions like curfews, as well as economic stressors were perceived as major drivers of violence. This study also suggested that men were particularly stressed by economic woes; this may have contributed to increased perpetration of violence. However, in the Nairobi study, participants pointed to disruptions in traditional gender norms as a driver of violence, and indicated that women earners whose husbands lost jobs may be more susceptible to violence (14). However, some participants in our study disagreed, suggesting that employed women may be more protected from violence—as women earners with jobless partners were thought to hold more power in their relationships.

Participants raised numerous potential factors contributing to rise in violence for AGYW—many of which were consistent with both the Socioecological Model and the Pathways Linking Pandemics & Violence Against Women and Children Model (2, 18). With regards to the Socioecological Model, AGYW described gender norms and expectations that both increased violence and reduced reporting. AGYW explained the occurrence of violence in their community as being at least partially a result of the pandemic's economic effects making jobs scarce, leading to rising unemployment and underemployment. Many of these economic effects were in part tied to the societal and structural restrictions put in place by the government to reduce pandemic spread. Both the isolation caused by these restrictions and the economic hardship for families were detrimental to interpersonal relationships and individual stress levels. Stress on individuals—particularly male family members—may lead to violence, or to substance use which, in turn, increased violence. Despite emphasis on economic issues driving violence, AGYW frequently discussed stress, mental health issues, and substance use as the final variables on the causal pathway which ultimately led to violence. Societal and government interventions such as pandemic lockdowns also made accessing care and community support services difficult—reducing interpersonal connections with guidance counselors and other mentors, and increasing AGYW vulnerability.

Many of our findings were also consistent with the suggested pathways to violence within the Pathways Linking Pandemics & Violence Against Women and Children Model (3). Economic insecurity and poverty-related stress was a consistent factor pointed to by participants as a pathway to violence. Although necessary to control viral spread, quarantines and social isolation increased violence directly in various ways—for example, by reducing women's ability to escape abusive partners and reducing women's ability to seek care. AGYW reported such “lockdown” measures also increased violence indirectly through their role in exacerbating mental health and substance use issues. Demographic shifts in population distribution may have also contributed to risk—AGYW reported that family structures shifted, and that some AGYW were forced to move in with different family members or out to rural areas, putting them at risk of violence and/or reducing their access to services. Reduced health services and community services also meant that women and girls were less able to seek help or support, and disintegration of referral systems meant AGYW were less able to escape abusive situations. Notably, AGYW did not discuss virus-specific methods of abuse, such as deprivation of masks or soap; however, as it is not a commonly-discussed type of violence, it is possible participants did not consider this as a form of violence or abuse. More prompting of participants may be necessary than was employed in this study to elicit information about this type of violence. This form of abuse was spontaneously discussed in a second branch of our study in which we conducted key informant interviews with service providers and community leaders working with AGYW. Lack of access to services and disruption of community referral systems was expanded on in detail within these key informant interviews; this will be the focus of a future report.

Consistent with the Pathways model, our study participants identified exacerbation of mental health and substance use as having a pivotal role within many pathways to violence. For example, when discussing economic insecurity, AGYW would frequently discuss poverty-related stress, mental health symptoms and substance use as major drivers for violence perpetration, and for creating situations where people were at risk of victimization. AGYW connected quarantines and isolation with violence through these viral control measures' effects on household income, mental health, and substance use. In terms of mental health, there was some consensus that the pandemic exacerbated mental health and substance abuse challenges of male family members, who then “released” this stress on female family members or strangers in the form of violence. Economic stressors could also directly lead to violence, such as causing parents to initiate early/forced marriages for their children, or leading AGYW to engage in transactional sexual relationships that became violent. In turn, AGYW felt that women's and AGYW's mental health declined.

Unique from the Pathways model was participants' focused attention on mental health as a unique driver of violence. Mental health is discussed within the Pandemic Pathways model, particularly with regards to social isolation and stress related to poverty. It is also noted that pathways may be exacerbated by declines in mental health (3). These were certainly areas of major discussion within our FGDs and areas in which our data is consistent with this model. However, AGYW spoke about and emphasized mental health and substance use both in relation to these topics and independently. Lack of mental health care access was discussed as a major challenge both in general, and with regards to preventing and stopping violence in their communities. It is important from a public health perspective to recognize the independent importance of mental health in violence prevention and intervention, both in general and given the global mental health crisis that emerged during the pandemic, particularly among adolescents (35, 36). As in many countries, Kenya's already under-resourced mental health system suffered from a shortage of human and financial resources that was exacerbated by a lack of dedicated funding or programming within the larger COVID-19 response (37). Despite increased rates of violence, services for survivors were difficult to access and scarce due to high demand, with mental health resources particularly inaccessible for vulnerable or low-income survivors (30). In an investigation of violence in Kenya during the pandemic, for example, none of the survivors identified were able to access the comprehensive healthcare they needed, and most received little or no mental health support (38). If we are to meet the needs of AGYW in future global health emergencies, it will require investing greater financial and human resources in mental health care, and recognizing the importance of treating and preventing trauma, mental illness, and substance abuse as a pivotal part of pandemic preparedness and response.

AGYW in our study also emphasized the lack of mental health resources and mental health support in their communities, and emphasized that this dearth of resources exacerbated violence. AGYW desired greater opportunities for interaction with mental health counselors, and wanted greater personal and community access to health and psychoeducation. It is unfair and incorrect to say that mental illness causes violence (39); however, a lack of mental health support in difficult times may contribute to violence through exacerbation of stress, substance use issues, and challenges in relationships, parenting, problem-solving, and other issues. Relatedly, investment in community mental healthcare more broadly may help prevent violence against AGYW through reduction in such risk factors (39). This has been shown in prior study: A randomized-controlled trial of a community-based intervention in South Africa found that improved caregiver mental health resulted in reduced caregiver substance use, improved household financial status, and reduced violence against children (40).

AGYW in our study identified several interventions they thought would be useful in the future, including economic empowerment, mental health interventions, and to a lesser degree, community education interventions. AGYW felt that economic empowerment interventions would help prevent trauma and violence, whereas mental health support was the most important intervention to stop trauma and violence once it was already occurring. These ideas from the AGYW in our study are consistent with recommendations from major international organizations and research on guidelines for violence prevention (41, 42). These provide potential ideas of important and acceptable interventions to consider when aiming to reduce violence in a peri-pandemic period in this population, and perhaps more generally. However, this is not an all-encompassing recommendation. Given their high costs and conceptual nature, AGYW were not asked to specifically comment on gender norms transformative interventions, but were interested in further education about various gender-related topics. Given our focus on providing services for AGYW, participants were also not specifically asked about a need for interventions addressing men and boys. It is notable, however, that some participants indicated a feeling of abandonment by community programs locally because they were not perceived as targeting or engaging with AGYW. Programs engaging men for gender equality have increased in popularity over recent years (43); it is important that such programs do not come at the expense of reduced attention to the needs of AGYW, and should engage with local AGYW to understand their specific needs.

While the landscape of COVID-19 response has changed drastically over the last few years, it has created a new reality. We are living not just in an era of climate change, but of pandemics, and must be prepared for the realities of future humanitarian emergencies and their effects on our most vulnerable populations, such as AGYW. This study provides insight into the experiences of a severely understudied population during the pandemic, and lessons learned should be considered when planning for future pandemics or other major emergencies. AGYW voices are essential in the creation and roll-out of pandemic preparation and response plans. Primary and secondary prevention of violence against women and children requires empowerment—through such measures as long-term economic and educational interventions, but also acute measures during disasters, such as direct cash transfers, rent assistance, supportive housing, generation of short-term employment opportunities to assist in relief, or other direct aid. Relatedly, reduction of extant violence against women and children and its deleterious effects requires action in tertiary prevention—holistic support for survivors, creating clear pathways to care, and increasing access to trauma-informed and trauma-focused mental health programs within communities (including substance abuse programming). Investing in such programs ahead of humanitarian emergencies will not only allow communities to build resilience, but will allow for disaster response that does more to meet the needs of the most vulnerable.

### 4.2. Limitations

Some limitations arise from our sampling strategy. Firstly, due to our project partners, our sample consists of AGYW exposed to GBV who were already connected with care. These AGYW may have baseline differences from the overall community and AGYW who are not connected with care; this idea is possibly supported by the fact that our sample is demographically different from the Kisumu County general population. For example, our participants are more educated and more likely to be single, perhaps indicating a level of economic or other privilege that may have increased connectedness with care that other AGYW did not have. This may result in differences in their needs, experiences, and desires for intervention after the pandemic, and may reduce our study's applicability within unreached populations. Relatedly, our study's population is primarily sampled within Kisumu's urban and peri-urban communities, which are better-resourced than its rural areas; due to pandemic limitations, we were unable to sample outside of Kisumu city. Further research would be required to determine if the experiences of girls in rural Kisumu matched that of girls in urban and peri-urban Kisumu. Perhaps both due to their pre-existing resources and to the resources that they have gained through being connected with care, participants may be more knowledgeable about topics such as gender norms and dynamics, gender-based violence, and mental health than typical AGYW from their communities. It is possible that given they may have been exposed to education surrounding violence and gender, AGYW may have already been made aware of concepts and interventions discussed in focus groups, and may have been speaking from places of knowledge rather than personal experience or opinion. This may limit the validity of findings.

Second, evidence prior to our study found that GBV in Kenya has shown shifting demographics, with, on average, younger girls exposed to violence than prior to the pandemic (11, 12). Nonetheless, our study only recruited AGYW age 15 and up. There were several reasons for this, discussed in section 2.3. One of these reasons was that younger girls may not have capacity to assent or may have trouble understanding the discussion and proposed interventions. This concern was corroborated and evidenced as a limitation of the study: 15–18 year-olds provided less concrete and rich data than their older counterparts. However, epidemiologically, there is great concern that younger girls have been affected by violence in Kenya with much greater frequency during the pandemic; it is likely that this study does not sufficiently capture the experiences and needs of these younger girls.

Perhaps due to the unique focus group environment and/or the sensitive nature of the topics discussed in the study, AGYW were sometimes delayed in opening up in discussions, with the older groups opening up more quickly than younger groups. Perhaps relatedly, transcripts from younger groups tend to reflect more reserved engagement. Younger AGYW may take more time to “warm up” within focus groups, and our younger group data may reflect a less open discussion than that of our older groups. In future studies, focus groups with younger participants may need to employ more creative question types or bonding time prior to initiation of the group, so as to prompt greater engagement in earlier questions in the focus groups.

AGYW sometimes spoke in hypotheticals (e.g., describing scenarios in the second or third person) when speaking about difficult topics. This could reflect how questions were phrased, as most questions were scripted to ask participants' opinions about AGYW in their community, as opposed to directly querying their personal experiences. As discussed in section 2.6, this style of question was purposefully employed so as to avoid revictimization/retraumatization. However, participants were recruited due to their personal experiences of GBV, and details and delivery of statements gave indication that AGYW were speaking from personal experience. This phenomena has been observed in other qualitative studies of this age group in Kenya (14) and may reflect stigma around violence, particularly when intrafamilial.

Lastly, some AGYW participants interpreted our descriptions of proposed interventions differently than intended—such as the FB. This may limit validity of findings surrounding discussion of proposed interventions, and indicates that as interventions are piloted, careful testing and community education will be necessary.

### 4.3. Future directions

The study participants revealed additional insights into AGYW experiences, youth mental health, violence, and trauma which merit additional research. For example, youth made frequent reference to suicide in relation to trauma experiences, which could indicate a higher risk profile for trauma survivors or cultural beliefs surrounding suicide or trauma. Further, AGYW understanding of the difference between sexual assault and sexual harassment, or lack thereof, may indicate a need for education, or experiential differences, and warrants research. Due to difficulty accessing populations, stigma, and slowly emerging global interest, both quantitative and qualitative research on such topics is sparse and much-needed.

Research examining the long-term impact of the pandemic on the mental health of AGYW is necessary, particularly as it relates to trauma and violence. Resource availability has shifted during the pandemic and changes have been made to service delivery; thus, the resource landscape and barriers/facilitators to care for violence, trauma and mental health are likely to change considerably in the coming years. Improved understanding through research will help equip populations to safeguard the wellbeing of AGYW in advance of the next global pandemic.

Despite the pandemic's negative impacts on both mental health and experiences of violence for youth worldwide (44), trauma-focused mental health interventions or those that address the experience of violence remain sparse, particularly in low- and middle-income countries (45). However, there is increasing recognition that in order for services to be effective for trauma survivors, they must be trauma-informed, or even specifically trauma-focused (46). Recognizing that truly trauma-informed approaches are requisitely empowerment-driven and require inclusion and participation of the communities they work with, it is our hope that this study can build upon a body of evidence to create better interventions that meet the needs of AGYW survivors.

## Data Availability

The datasets presented in this article are not readily available because the data are not publicly available as they contain information about sensitive topics such as violence from a population of youth (in some cases, minors) that could compromise the privacy of research participants. The data that support the findings of this study are available on request from the corresponding author, with proof of appropriate IRB approvals and human subjects training. Requests to access the datasets should be directed to CS, cleas@stanford.edu.
